# User Experiences of Transitioning From a Homegrown Electronic Health Record to a Vendor-Based Product in the Department of Veterans Affairs: Qualitative Findings From a Mixed Methods Evaluation

**DOI:** 10.2196/46901

**Published:** 2024-09-10

**Authors:** Ekaterina Anderson, Megan Moldestad, Julian Brunner, Sherry Ball, Christian Helfrich, Jay Orlander, Seppo Rinne, George Sayre

**Affiliations:** 1 Center for Health Optimization and Implementation Research Veterans Affairs Bedford Healthcare System Bedford, MA United States; 2 Division of Health Informatics and Implementation Science Department of Population and Quantitative Health Sciences University of Massachusetts Chan Medical School Worcester, MA United States; 3 Veterans Affairs Seattle-Denver Center of Innovation for Veteran-Centered and Value-Driven Care Veterans Affairs Puget Sound Health Care System Seattle, WA United States; 4 Veterans Affairs Center for the Study of Healthcare Innovation, Implementation & Policy Veterans Affairs Greater Los Angeles Healthcare System North Hills, CA United States; 5 Veterans Affairs Northeast Ohio Healthcare System Cleveland, OH United States; 6 School of Public Health University of Washington Seattle, WA United States; 7 Chobanian & Avedisian School of Medicine Boston University Boston, MA United States; 8 Veterans Affairs Boston Healthcare System Boston, MA United States; 9 The Pulmonary Center Chobanian & Avedisian School of Medicine Boston University Boston, MA United States

**Keywords:** electronic health records, United States Department of Veterans Affairs, Veterans Affairs, organizational change, delivery of health care, integrated, medical informatics

## Abstract

**Background:**

The Department of Veterans Affairs (VA), the largest nationally integrated health system in the United States, is transitioning from its homegrown electronic health record (EHR) to a new vendor-based EHR, Oracle Cerner. Experiences of the first VA site to transition have been widely discussed in the media, but in-depth accounts based on rigorous research are lacking.

**Objective:**

We sought to explore employee perspectives on the rationale for, and value of, transitioning from a VA-tailored EHR to a vendor-based product.

**Methods:**

As part of a larger mixed methods, multisite, formative evaluation of VA clinician and staff experiences with the EHR transition, we conducted semistructured interviews at the Mann-Grandstaff VA Medical Center before, during, and after going live in October 2020. In total, we completed 122 interviews with 26 participants across multiple departments.

**Results:**

Before the new vendor-based EHR went live, participants initially expressed cautious optimism about the transition. However, in subsequent interviews following the go-live, participants increasingly critiqued the vendor’s understanding of VA’s needs, values, and workflows, as well as what they perceived as an inadequate fit between the functionalities of the new vendor-based EHR system and VA’s characteristic approach to care. As much as a year after going live, participants reiterated these concerns while also expressing a desire for substantive changes to the transition process, with some questioning the value of continuing with the transition.

**Conclusions:**

VA’s transition from a homegrown EHR to a vendor-based EHR system has presented substantial challenges, both practical and cultural in nature. Consequently, it is a valuable case study for understanding the sociotechnical dimension of EHR-to-EHR transitions. These findings have implications for both VA leadership and the broader community of policy makers, vendors, informaticists, and others involved in large-scale health information technology implementations.

## Introduction

### Background

Driven by the Health Information Technology for Economic and Clinical Health Act and other policies and incentives [1], in the past 15 to 20 years, health care organizations in the United States have almost universally replaced paper-based health record systems with electronic health records (EHRs) [2,3]. Fueled by ever-increasing technological embeddedness in health care, transitions from one EHR to another have become more common [4]. These transitions are in part driven by the EHR vendor market that is undergoing rapid consolidation [2,5], as well as health systems’ own quest to improve the efficiency and quality of care delivered. Notwithstanding the wave of paper-to-EHR implementations that gave rise to a rich body of literature [6-10], research on EHR-to-EHR transitions has so far been limited [11,12], producing relatively few qualitative case studies that explore leadership or employees’ experiences and perspectives in depth [13,14]. This lack of literature limits the ability of health care organizations to learn from their predecessors’ experiences when attempting similar large-scale health information technology (HIT) implementations.

In 2020, the Department of Veterans Affairs (VA) began a multiyear “rolling wave” replacement of its homegrown EHR with a vendor-based product. VA is the largest nationally integrated system in the United States, and a sweeping undertaking like the current EHR transition is an inherent matter of public interest. The EHR modernization has already affected—and will continue to affect—the ability of VA to provide high-quality, safe, and timely care to the veterans that it serves [15]. The transition also has direct implications for the morale and organizational commitment of VA’s considerable workforce. Finally, the cost of the EHR modernization, originally estimated at US $10 billion [16,17], is now projected to reach US $50 billion over the next decades [18-20].

The VA has a long history of using information technology (IT) to meet the health care needs of veterans. VA’s computational infrastructure [21] comprises the Veterans Health Information Systems and Technology Architecture (VistA) and VistA’s interface, the Computerized Patient Record System (CPRS). VistA/CPRS have their origins in the late 1970s, when a group of doctors and IT staff at VA began developing HIT solutions independently of the national office [22]. This approach led to a highly decentralized system where each individual HIT product was tailored to provider and staff workflows and practices, including programs common in current commercial EHRs but revolutionary at the time, such as scheduling tools, mental-health assessments, and provider order entry systems [22,23].

In the 1990s, after decades of increasing complexity within the system, VA underwent sweeping systemic change to improve care quality, service, and overall operations, including upgrading the HIT infrastructure to ensure connectivity across the enterprise [23,24]. As part of this extensive series of reforms, VA moved to implement VistA/CPRS as a national-level, integrated EHR, replacing site-level EHR systems that had been developed by local informaticists in close collaboration with clinicians and administration. VistA/CPRS has demonstrated high rates of user satisfaction [25] and, as a publicly available system, has been adopted by over 30 health care systems worldwide [26].

In early 2017, then VA secretary David Shulkin announced that VistA did not have the infrastructure, security, or interoperability required for its continued use [27]. Shulkin and other top-level VA officials advocated for a single electronic medical record that would follow servicemembers from active to retired service, enabled by an EHR system shared by the Department of Defense (DoD) and Veterans Health Administration (VHA). In June of 2017, Shulkin announced his decision to move forward with adopting what was described as a next-generation EHR system [28]. The Veterans’ Electronic Health Record Modernization Oversight Act (2017) directed VA to provide Congress with documentation of the effort’s progress and costs. In May of 2018, then acting VA secretary Robert Wilkie announced that the VA had signed a US $10 billion contract with Cerner Corporation and that VA would transition to the Cerner EHR (referred to under its current name of “Oracle Cerner” in the rest of the paper). In June of 2018, the VA established the Office of Electronic Health Record Modernization, an office whose focus was to ensure a successful transition from VA’s legacy EHR to Oracle Cerner. The DoD began Oracle Cerner deployment earlier that same year.

The decision to switch from the VA’s homegrown, legacy system to a commercial EHR sparked conflicting views within the VHA as well as in political circles. Proponents argued the move would bring the federal health care system up to date with other health care systems, reduce costs, and support interoperability with the DoD records, thus providing servicemembers with a seamless health care experience when they transitioned to VHA care [29,30]. Opponents criticized the no-bid contract awarded to Oracle Cerner [30], citing CPRS’s ongoing positive ratings by physicians as being more useful than commercial systems, more provider and staff friendly, and customized for VHA use [25,31]. Despite these reservations, the VA proceeded with the transition.

### Objective

In this paper, we report on the perspectives of employees at the first VA site to undergo the EHR transition—Mann-Grandstaff VA Medical Center (MGVAMC) in Spokane, Washington. We focus, specifically, on employees’ diverse and shifting perspectives regarding the rationale for and value of transitioning from a homegrown EHR that was custom-built for VA and highly tailored to its organizational culture and processes to a vendor-based product. The inaugural site’s experiences, widely reported on in the press [32-35], may have already set the tone for how VA’s EHR transition is perceived by VA’s frontline employees, leaders, and the public—becoming a benchmark for subsequent sites to compare themselves against. We believe that it is important to add nuance to this discourse by reporting on the first-hand employee perspectives that were collected using rigorous methods during the course of the transition. By presenting a case study from one of the largest and most influential health systems in the United States, we seek to contribute to the growing literature on the dynamics, challenges, and impact of EHR-to-EHR transitions.

## Methods

### Research Design

Since 2020, our team of VA researchers and clinicians with interdisciplinary expertise in implementation science, quality improvement, informatics, and organizational change has been conducting a concurrent mixed methods, multisite, formative evaluation to understand VA clinician and staff experiences with the EHR transition [36-38]. Our methods and findings are described in line with the COREQ (Consolidated Criteria for Reporting Qualitative Research) [39], with additional information provided in Multimedia Appendix 1.

### Setting

For this manuscript, we report on the qualitative data obtained between September 2020 and November 2021 at MGVAMC as the first VA site to undergo the EHR transition, with the go-live on October 24, 2020. With 1354 full-time employees, MGVAMC and its associated community-based outpatient clinics provide primary and secondary care to approximately 35,000 veterans in the Spokane area of Washington. The main campus includes a 36-bed acute care hospital and a 34-bed community living center (a VA term for a facility that provides subacute rehabilitation and long-term care, including hospice care at some VA Medical Centers. Additional outpatient care is available at associated sites (2 multispecialty, 2 rural, and 1 mobile clinic) [40].

### Recruitment and Data Collection

We used a snowball sampling approach to recruit participants. Several months before “go-live” (the date on which all staff would begin using the new system for patient care), principal investigators (GS and SR) held multiple stakeholder engagement meetings with local leaders to identify groups of individuals who routinely work together across clinical services that may be willing to participate. During subsequent interview rounds, participants were asked to identify additional individuals for our team to approach.

Semistructured interviews were used to explore end user EHR transition experiences. The interview guide (Multimedia Appendix 2) was iteratively developed to include open-ended, nonleading questions to capture rich data on domains of interest, including EHR usability, EHR training and support, impressions of the VA’s decision to switch to Oracle Cerner, employee burnout, and well-being. The interview guide was pilot-tested with staff members at a different site that was initially supposed to go live, but the decision was reversed. We interviewed participants at multiple points in time: before go-live (September 2020), during go-live (October 2020), 2 months after go-live (December 2020), and 10+ months after go-live. Interviews conducted during go-live (“check-ins”) were kept brief (approximately 15 min) and focused to minimize the burden on participants. The remaining were approximately 60 minutes in length. Interviews were conducted by team members in both principal investigator and staff roles over the phone or Microsoft Teams (Microsoft Corporation), due to the public health emergency restrictions related to the COVID-19 pandemic at the time. The interviews were recorded and transcribed verbatim. Interview guides were modified iteratively for clarity and to expand the focus of exploration, reflecting emergent data. Whenever possible, participants were paired with the same interviewer across data collection points to increase comfort, consistency, and trust. All interviewers were trained in qualitative research and semistructured interviewing. Following each interview, interviewers completed notes summarizing the interview content and capturing initial reflections. These notes were subsequently referred to during team discussions to inform analysis.

In total, we completed 122 interviews with 26 individuals, including providers, nurses, and other clinical staff (eg, medical support assistants), as well as a smaller number of clinical administrators and leaders across multiple departments and disciplines at MGVAMC (Table 1).

**Table 1:** Participants and data collection (interviews: n=122; total participants: n=26^a^).

	Stakeholder engagement meetings (n=11), n (%)	Prego interviews before go-live (n=21), n (%)	Check-ins (n=47), n (%)	Interviews after go-live (n=23), n (%)	Interviews 1 year after go-live (n=20), n (%)	Total, n (%)
Leadership and providers^b^	11 (100)	11 (52)	22 (47)	14 (61)	13 (65)	71 (58)
Nurses^c^	0 (0)	6 (29)	12 (25)	4 (17)	5 (25)	27 (22)
Staff^d^	0 (0)	4 (19)	13 (28)	5 (22)	2 (10)	24 (19)

^a^Only the total sample size provided as a more specific breakdown of how many individuals within each participant category were interviewed at each stage may endanger participant anonymity given the site’s small employee body.

^b^Physicians, clinical pharmacists, and psychologists.

^c^Registered nurses and licensed practical nurses.

^d^Medical assistants, phlebotomists, counselors, audiologists, and physical therapists.

### Data Analysis

We initially used deductive and inductive content analysis [41], followed by reflexive thematic analysis [42,43] to generate a more in-depth understanding of the data. The full team first generated a list of a priori categories reflecting the project’s aims (eg, impressions of the VA’s decision to switch to Cerner, EHR support, EHR training, software functionality, and impact on veterans). A subset of 8 team members conducted line-by-line coding using a qualitative data analysis software program (version 9, ATLAS.ti; ATLAS.ti Scientific Software Development GmbH). New codes and code groups were added throughout the coding process to reflect emergent concepts that did not fit the existing schema. Analytic memos reflected coders’ impressions of patterns and contrasts in the data. The coding team met weekly to troubleshoot issues with the coding process logistics and discuss emerging analytical insights.

During these meetings, we identified topics warranting in-depth exploration, which included participant reflections on how well the VA’s homegrown EHR versus the commercial one fit their practice and VA’s needs as an organization. At that point, the first author conducted an iterative review of relevant code categories and codes, as well as reread individual interviews to better understand the context in which the Oracle Cerner versus VistA/CPRS contrast was invoked and identify the range of experiences. After the preliminary themes were generated and outlined by the first author, the themes were then iteratively refined with input from the full team of coauthors. In line with an inductive, critical, constructivist approach to reflexive thematic analysis as defined by Braun and Clarke [44,45], we avoided imposing a theory on our themes and closely attended to the discursive and rhetorical means through which participants constructed meaning around their experiences. We reached inductive thematic saturation [46] in the sense that no new codes or themes emerged during the final analytic stage.

### Ethical Considerations

The VA Bedford Healthcare System Institutional Review Board designated this evaluation as nonresearch quality improvement, that is, not subject to the institutional review board oversight. During recruitment, potential participants received information about the evaluation’s objective and methods, their right not to participate without any repercussions, as well as their rights as participants, including the right to withdraw at any point. Before the initial interview, participants provided verbal consent, which was audio recorded. Participants were asked to confirm their willingness to participate and reminded about the option to withdraw at each subsequent interview. In accordance with VA policy on privacy and data security, interview recordings, transcripts, and notes are kept behind a firewall on a secure VA server and only accessible to the project team to protect the participants’ privacy and confidentiality; all files were assigned code names that could not be traced to individual participants. Participants were not compensated for their participation in this evaluation, as VA rules prohibit VA employees from receiving compensation for participating in VA research or quality improvement while on duty.

## Results

### Overview

Here, we present 4 themes that capture end users’ experiences across time: cautious optimism about the new commercial EHR, tempered by apprehension; disappointment with the vendor’s limited understanding of the VA; the tenuous fit between the new EHR and VA’s needs; and desire to change course. Themes are supported with illustrative quotes. For each quote, we provide a 3-part ID consisting of the nonidentifiable participant number, their general role (provider or leader, nurse, or staff), and the timing of the interview (with “pre” referring to interviews before go-live, “during” for briefer check-ins conducted during the go-live, “post” for interviews done 1-3 months after the go-live, and “10 m” for interviews done 10-12 months after the go-live).

### Cautious Optimism About the New Commercial EHR, Tempered by Apprehension

Before go-live, participants rarely invoked the homegrown versus vendor-based EHR dichotomy; instead, they primarily contrasted the new and the old EHRs as outdated versus modern. Specifically, some participants expressed a sense of cautious optimism about the upcoming transition to the new EHR, citing both what they perceived as outdated features of the VistA/CPRS system and the attractive characteristics of its more modern replacement:

We [VA] had the first electronic health record, which was great. But now it’s the oldest electronic health record, and it hasn’t been updated, and it’s clunky.

P18_Provider_Pre

There’s lots of layers to do one task [in CPRS]. And then do not even get [me] started on the current scheduling system, the current scheduling system is horrible. Horrible. ... I’m not sure if everybody’s pleased that it’s going to be Cerner, but I feel like everybody is pleased that it’s going to be switched.

P17_Provider_Pre

I’m glad that we’re finally going to something that’s more modern and user friendly.

P23_Nurse_Pre

For a few of the participants who were positive about the transition, the outdated versus modern dichotomy even offered a language for setting themselves apart from colleagues who they perceived to be less forward-looking and more resistant to change:

The VA, in my humble opinion, has a bunch of dinosaurs. ... The 15 year plus employees...like what they were doing, they’re comfortable with what they were doing, change is bad. ...And then there’s the newer employees that are like, ‘come on, man, let me help get this taken care of.

P18_Provider_Pre

I wish more people were a little bit more openminded, you know, with, ‘hey, we’re doing it, let’s see how it goes.’ Rather than a ‘bah humbug’ type of an attitude. That would be helpful. But nobody could control that but each individual. ... But I’m excited.

P15_Staff_Pre

However, even these early accounts contained some apprehension about the change to a new EHR system. Some expressed doubts about the quality of the new EHR itself. For example, one participant endorsed the need for modernization yet took issue with the very assumption that Oracle Cerner would constitute a more “modern” alternative to CPRS:

I think the decision to modernize is good. ... So, I think that was a good decision and a long time coming. Personally, Cerner, especially now that I’ve actually seen it...I would say to me it looks very outdated as well. ... To me, I’m not really impressed with it so far. ... But I’m sure, obviously, there’s so much that went into that. So, so far not overly impressed with Cerner specifically, but we’ll see.

P5_Provider_Pre

In contrast to this general criticism, other participants raised their concerns about the ability of the new EHR to support VA’s established roles and practices:

I know [Cerner’s] a great accounting system, and all of the coding stuff, but how do I do what I need to do? I don’t really care about how somebody else gets through Cerner...

P21_Provider_Pre

[In CPRS], we probably have a couple dozen templates that make documentation a little easier.... I do feel like that’s going to be a bit of a switch when we go to Cerner, because it sounds like they really don’t have any templates, or very minimal templates.

P17_Provider_Pre

In sum, while before going live, some participants saw the new EHR as a more modern alternative to the older EHR and a potentially good option for VA, the concern about the new product’s ability to meet VA’s particular needs was noticeable even at this early stage.

### Disappointment With the Vendor’s Limited Understanding of the VA

As go-live drew near and then came and went, participants voiced concerns that the vendor was insufficiently knowledgeable about and attentive to VA’s manner of providing care, as in the following example before go-live:

I really don’t know how much information (Cerner) had about what different services did, and it felt to me in the initial stage like...they were just giving us their stuff with no sense that VA care is very different than care in the community or private care. But I think it’s better now.

P14_Provider_Pre

Following go-live, this concern about the vendor was expressed much more forcefully by a few participants. In the example here, the participant—perhaps anticipating the “some people don’t like change” critique—implied that the vendor not doing its due diligence to learn about VA contributed to difficulties that went beyond the regular level of “hardship” that is to be “expected” during EHR transitions:

...there’s hardship that should be expected with any transition. I just...question the understanding of the complexity of the VA system prior to launch.

P13_Provider_Post

The sense of disappointment with the vendor’s understanding of VA often came up in the context of training. Many participants felt that the training in the new EHR was poorly or not at all tailored to VA’s needs. One specific complaint, expressed by several participants, was that Oracle Cerner trainers were unaware of the nuances of various roles in the VA:

Having someone that can really train you in accordance to your specifications of your job would be better. ... Because the Cerner trainers said, ‘I don’t know what you do. This is what I know about the system.

P24_Provider_Pre

While the insufficient number of vendor training staff with a clinical background was invoked by some participants as the root of the training problems, even having a trainer with a clinical background was not necessarily a panacea if they were not familiar with VA’s context:

They should have had providers training providers, nurses training nurses, etc. Instead, they had people with really no medical background that were just computer people, that did not have a good sense of day-to-day clinic flow and things like that, and really were not able to answer any of the questions I had. ... But I know even [Cerner’s trainer with a physician background] was pretty frustrated, because he was just like, ‘I’ve never seen it like this before, why is the VA system so weird...

P5_Leader_Post

Some participants also conjectured that trainers had a poor grasp of VA’s workflows and were thus unable to draw CPRS-to-Oracle Cerner parallels or analogies that VA employees would likely have found useful while learning to use the new system:

...a lot of the issue was the people that built Cerner for our VA System had never even opened CPRS. So, there’s a lot of things where we’ll say, ‘how would you do this in CPRS’? And they don’t know what we’re talking about, because they don’t know what CPRS looks like. So, I feel like if there had been input... how Cerner can be modified to what we need for our workflow, that would’ve been helpful along the way.

P8_Nurse_Pre

For a few participants, the lack of attunement was also seen in the language gap between the vendor and the VA. Participants described divergences between how the same or similar operations or functions are named in *CPRS versus Oracle* Cerner—for example, “consult” (CPRS) versus “referral” (Oracle Cerner)—pointing out that the similarities between the 2 were poorly clarified:

...nobody gave us a language. We didn’t learn how to speak Cerner, and that’s what should’ve come first.

P6_Provider_During

Reflecting on the trajectory of the EHR transition almost a year following the go-live, one participant highlighted both the quality of training and the lack of understanding of VA’s approach to care embodied in the EHR itself as 2 main challenges—a sentiment that aptly provides a bridge to the next theme:

[What made things difficult is] the fact that Cerner didn’t seem to take the time to really understand the work we do, and then be able to wed their product to our work. And then having training that was completely abysmal. These were all things that made it a disastrous implementation in my opinion.

P1_Leader_10M

### The Tenuous Fit Between the New EHR and VA’s Needs

#### Overview

In addition to taking issue with the vendor’s degree of familiarity with VA’s context, many participants also questioned the fit between the functionality of the Oracle Cerner EHR and VA’s needs. Some of these statements were general in nature. For example, several participants complained that Oracle Cerner was poorly suited to helping them complete their daily workflow, as in the following example from the participant who characterized the new EHR as unintuitive:

I am still struggling with this program. ... There’s a lot of things that I still don’t know how to do that are basic functions of my job that aren’t built yet and not working well...I’m seeing...about 5 patients a day, I work 10-hour days. And it’s not easy, and I am putting in extra time to try to get everything done, but it’s happening. Do I like it? No. I think this program...is very not intuitive. It doesn’t make any pathway sensible to me or easy, I have to follow a list of steps still for the things that I’m not doing five times a day. And, I’m really surprised VA bought this thing, but here we are. ... If they’re expecting it to save money or make things easier, I think they got sold a pig in a poke, that’s kind of my 2 cents.

P14_Provider_During

Another participant made a similar claim, pointing to the paradox of the “older” EHR (CPRS/VistA) being both simpler and more efficient than the more “modern” Oracle Cerner:

...they should’ve sat with people that were really experienced and seen exactly what they do every day...And how simplified [CPRS is] compared to this thing that they built...if they sat with...an experienced end user for a week, I think their eyes would’ve been open as to the amount of work that is done by someone in my position. Or even a provider. And each person, just to see exactly what is done and how efficient they are with that old program, and how streamlined, believe it or not it was, even though it was old, compared to this one. Because we’ll tell you, get ready for a lot of clicks.

P10_Staff_Post

Notably, however, we recorded numerous versions of the idea that Oracle Cerner may be a poor fit for VA as an integrated delivery system with robust interdisciplinary coordination and the ability to provide wrap-around services for a clinically and socially complex patient population. A few interviewees explicitly juxtaposed VA and those health systems where billing is more central to workflows, arguing that Oracle Cerner is a better fit for the latter:

...I don’t think that it was looked at strongly enough, whether it would be compatible with the VA or not. It’s a system that...works well in...money-making systems, where you need to link everything to an encounter for compensation purposes, and VA doesn’t work like that.

P5_Leader_10M

So, Cerner is an off-the-shelf [EHR]. The VA is not [a] ... for-profit doctor’s office or hospital system. It’s its own Garanimal. And it is not malleable. It is not massageable. ... the VA makes us have to do things a certain way, accomplish certain goals, and really work at lickety split speed with precision.

P6_Provider_10M

Several specific concerns about the fit between the new EHR functionality and VA’s needs were also discussed, as outlined in the following paragraphs.

#### Documenting the Work of Care

Many participants expressed concern about additional discrete steps for documenting care, that is, tasks done during and between formal appointments. This concern was raised primarily in the context of the requirement—absent in VistA/CPRS yet present in Cerner—to document “between-visit encounters” (eg, any interactions related to veteran care happening outside of appointments, such as phone calls or secure messages) as “encounters,” as opposed to simply adding notes to the veteran file. Multiple frontline staff participants complained about the requirement to create between-visit encounters as confusing and cumbersome. Interestingly, opinions as to *why* the between-visit encounter functionality created so many difficulties diverged. One participant took issue with the very idea of capturing the work done between veteran appointments as “encounters,” portraying it as a time-consuming requirement that was better suited to private health systems:

So, what the ‘between visit encounter’ is...in the world of generating income for the private practice...they want to account for every minute of every day what you’re doing ... We now have to do all of this work...to show what we’re doing with our time.

P6_Provider_During

A different participant with a leadership role opined that the between-visit encounter functionality was not problematic per se, as it provided a manner for more accurately capturing and providing credit for employees’ workload, yet acknowledged that *how* that functionality was set up was deeply flawed—slow and poorly automated:

The goal is good, they’re trying to capture the work people do between visits, because there’s a ton of work done for patients between visits. But the process just is not good. It needs to be a smart system that knows who you are, because the system knows who you are, and it...should be able to generate a pop-up that tells you that you need to generate a new encounter, and then just say, click here, and click, and then you have a new encounter. But right now it’s just this dumb system that isn’t smart, it doesn’t help you at all.

P2_Leader_Post

Finally, another participant, also with a leadership role, echoed the idea that the Cerner between-visit encounter functionality was set up in an inefficient manner. This participant provided further insight by arguing VA care involves a particularly large amount of workload done between formal appointments and that this fact was poorly understood by the vendor. The participant described their successful efforts in advocating for greater clarity around the new workflow while acknowledging that it still left much to be desired:

...for some reason, Cerner was not able to truly determine how we do our work and be able to create an [EHR] that allows us to do that work smoothly... I’m sure commercial clients are very different, [but] the VA has a very specific way of doing things...because of the way we do things in PACT (patient-aligned care teams), we might not see the patient for a year, and then there’s a ton of stuff that goes on within that year, but it’s not in the form of a visit. So they’re like, ‘ok, so we have to create this between visit type of encounter so that we can capture that work...’ ... So we got this very confusing kind of workflow about how you create this between visit encounter...I really pushed on hard to get them to figure out, what are we supposed to do here...at first...it was not even clear to the various change management and solution experts in Cerner, which is a little disappointing. So, we finally got to the point now where we kind of get when and how to do it, but it’s still terrible.

P1_Leader_Post

The difficulties of documenting care were mentioned in other contexts as well. For example, one participant explained that veteran appointments in the VA often have multiple components, combining visits with different professionals—to accommodate veterans who may want to have multiple problems addressed on the same occasion after traveling long distances. This participant indicated that Cerner makes multicomponent appointments more cumbersome by requiring different providers to “check in” and “check out” the Veteran for each new element of their outpatient visit. Interestingly, the participant attributed this phenomenon to Cerner being supposedly built for “inpatient” settings—another example of our participants offering their opinions about reasons for the strained fit between the new EHR and VA:

The reason it’s a problem is because Cerner was built as an inpatient product. They are an inpatient facility product. Outpatient operates differently, especially within the VA, right? ... Here, they come in and they drove 3 hours, so they’re going to get their eyes tested, they’re going to get their ears tested, and they want to see their PT and maybe the Mental Health. ‘Oh, and by the way, I may as well get my shots because I drove 3 hours to get here.’ ... That’s problematic, because... now we have to watch it, so they have to make sure that they check him out. Check him in for the first appointment, then check him out, then check him in for the second appointment, and check him out. And then check him in for the third appointment if he has a third one. It’s hairy.

P18_Provider_During

#### Communication and Coordination

Another area where the new EHR was perceived as inadequate for VA was in facilitating interprofessional communication and coordination within and across teams. VA facilities typically offer a substantially broader array of specialized mental health and social support services than other health systems, with extensive programs for posttraumatic stress disorder and other conditions with high prevalence among veterans [47,48]. The provision of such programs is facilitated by close interdisciplinary communication and coordination within and across facilities, as well as between the VA and community care providers. One participant suggested that VA’s homegrown EHR enabled robust and continuous multidisciplinary cooperation to a much greater extent than Cerner’s EHR did:

I do not think Cerner is right for the VA...with the VA, [veterans] have, like, their annual, and in between we have such great ancillary services. ... So sometimes even these very medically complicated patients may only need to see their Primary Care Provider once or twice a year because they have all of these great ancillary services. And...there’s constant communication back and forth between those services. ... And in CPRS, that’s a lot of back and forth on one chain. ... In Cerner, those create new between visit encounters every time. It adds another document, it’s just kind of jumbled in there. ... So, our way of communicating, the way we do it, doesn’t work ... [Cerner’s] more [of a] production-based system. And...that’s just not how the VA works.

P5_Leader_10M

Another aspect of Cerner seen by some as inadequate in comparison with CPRS/VistA was the limitations it sets on interprofessional collaboration *within* teams—a prominent feature of VA’s approach to care—due to the notable interface differences for users with different roles:

Everybody is somewhat annoyed by that because we all do not have the same pages that we are looking at. ... And it does not allow us to be able to collaborate and help one another in the way that we used to. Because it’s all specific to service lines. That’s a little frustrating. ... we’re collaborating less...we’re talking about clients—veterans—less holistically and working together as a team [less] because of the way this chart is set up.

P9_Provider_10M

#### Chronic Care and Population Health

Finally, we found references to Oracle Cerner having inadequate functionalities to support providing care to VA’s patient population, which was perceived to have a large proportion of individuals with multiple complex chronic conditions. For example, one participant felt that VA’s version of Oracle Cerner exposed this medically vulnerable population to potential harm or delayed care:

[There are] orders that went where they shouldn’t have gone or orders that didn’t go where they should’ve gone. ...We think that it’s essential for patient safety that if you got an order that went somewhere it shouldn’t, or didn’t go somewhere it should’ve, that it actually gets found and gets corrected or acted on. We have patients who are a lot more vulnerable (than DoD patients). They’re older, [have] multisystem failure, and not everybody is older with multisystem failure, but we certainly have a much larger portion of that than the DoD does.

P25_Leader_10M

On a related topic, one participant expressed concern with the poor functioning of registries to keep track of veterans with chronic health issues—a historically important feature of VA’s approach to population health that enables VA employees to review various indexes of health in their patient population to identify and proactively contact veterans with specific health care needs who may need care but may not otherwise seek it:

I know the nurses are struggling with the registries right now, trying to catch back up with those, and how it’s laid out. ... they’re having a hard time catching back up...that was something I know we were always really on top of, I know my team was. We always were really on top of patients that had chronic health conditions like diabetes, like hypertension, of keeping track of those. So that’s been a frustration from the nursing side of trying to play catch up with that and trying to get the correct information with that.

P16_Staff_10M

#### Desire to Change Course

In the interviews done after go-live—and especially 10 to 12 months later—participants continued to share reflections on the perceived inadequacy between the new EHR and VA’s needs. In addition, participants started to raise concerns about the future course of the EHR transition and reflect on the lessons learned from their experience as the first site.

Several participants invoked and dismissed the idea that continued difficulties experienced at their site may be due to poor training of staff members. Instead, they asserted that the EHR itself was flawed due to its insufficient tailoring to VA’s context. One participant noted feeling especially affronted given that Cerner had asked for input early on, only to seemingly ignore it in the actual build:

I personally do not feel as though we need any additional training. We have been trained on how to utilize this system. The system is just crappy. That’s the bottom line. ... The biggest, to me, kind of slap in the face is knowing that we had Cerner techs come and sit side by side with people for several months as we discussed, ‘hey, this is what the current CPRS charting system does, and this is what we don’t like, and this is what we would like for it to do.’ And, I mean, it’s just like, did they use any of that information when they created this charting system? I mean it’s really just ridiculous.

P9_Provider_10M

A few individuals went so far as to suggest that the decision to adopt Cerner was a mistake and that VA needs to go back to CPRS/VistA:

They need to take it back, and this is the reason why. Cerner is not a system that can be molded to the VA. And the VA is not a system that could be molded to Cerner.

P6_Provider_10M

... if I really could tell you what I really wish, I wish that the American people and Congress would say, this was unfortunate that we dumped this much money into this program, but I think they need to scrap it. I actually think that this program is not the right program. I don’t think it’s good for our Veterans, and I don’t think it’s good for our workers. ... CPRS was 100 times better than this. That’s what’s sad.

P17_Provider_10M

For others, however, the solution was less radical and involved strengthening VA staff’s ability to make necessary changes to the EHR system. One participant, who had a relatively positive view of the new EHR and the EHR transition, pointed out that, while most of what they perceived to be major problems with the new system had been resolved, staff lost communication channels for addressing any new issues they might encounter in the process of becoming more proficient in the new EHR:

The main issue that we are having now is the big problems that we had initially were all addressed and fixed, or they are in the process of being fixed, but now that all the urgent work stoppage type of issues have been resolved, all of our other issues, there’s no one to contact about them. Or we are not getting any kind of follow up when we do send in tickets. ... So I don’t really know what the solution is for that. ... Because as we get more familiar with the system, we’re going to have more issues that we didn’t know existed before, or things that we would like customized, but we just don’t know where to go with all of that information.

P8_Nurse_Post

Another participant explicitly framed going back to the homegrown system as impossible and undesirable yet argued that the vendor should be responsible for improving its product, with substantive input from frontline employees in the decision-making:

I think to have invested so much already financially, mentally, physically, emotionally...that there’s no going back...we still need to be involved in advanced electronic health records. But it’s kind of like, until they get this stuff taken care of. ... It’s just continuing to add to workload, frustration, people leaving. ... Get it done right, not [at] the expense of the government. At the expense of the company. ... And a lesson learned, but at the highest echelon where this was determined, that’s where you need that committee of individual actual worker bees to be involved in the decision-making. Or at least listened to and a part of the decision-making.

P19_Nurse_10M

Another participant echoed the need for greater accountability of the vendor to the VA, yet also attributed some of the difficulties with implementing the necessary changes to the laborious, multistakeholder decision-making process:

I feel like it’s basically been...VA people trying to come up with solutions. And there may or may not be Cerner people on calls when we’re talking about issues. Sometimes it seems like we just come up with these workarounds, and no one is holding Cerner to fix the problems. And some of that may be just because of the structure that VA created with this, where we have these national councils and then all of these agreements where it has to match between DoD and VA. So it just becomes this huge bureaucratic black hole. So, if we identify that there’s an issue, everybody has to agree that it’s an issue and that we need to find a solution, and then we have to agree on the solution.

P3_Leader_10M

Interestingly, not all participants supported the view that the new EHR can or should be perfectly tailored to VA’s needs. For example, one participant argued that the vendor needs to have better awareness of how the homegrown system works to be able to warn VA’s superusers about concrete workflow differences that might create challenges for the frontline staff:

...I never said [to Cerner], ‘you need to understand what we do so you can build your [EHR] to suit it.’ It was more like, we need to see what the [EHR] is so we can understand how we have to change our workflows. But that should’ve been done in advance so that at go live you have people, super-users, you know, supervisors, people like that, who actually can say, look, this is how we used to do it in CPRS, this is how we have to do it now. And that goes way beyond ‘here’s how you order something’ and ‘here’s how you write a note.’ These are like, you know, higher level workflows that they had no idea about...but someone has to know...so we could teach people how to do it, and how we’re going to get things done at go live. Not figure it out at go live.

P1_Leader_Post

## Discussion

### Principal Findings

Our findings tell a story of end users’ initial cautiously optimistic attitude toward transitioning to a new EHR, giving way to a concern that the vendor did not understand VA’s needs, values, and routines and a perception that the new EHR did not fit the VA’s approach to care, consistent with the results of a general survey of Mann-Grand staff end users [37]. Moreover, a year after go-live, participants reiterated these concerns and expressed a strong desire for substantive changes to the course of the EHR transition, although they differed in their preference for the nature of these changes. In addition to reporting on a momentous development within the largest integrated health system in the United States that is important in its own right, our work also contains important implications for health-system leadership, EHR vendors, researchers, and other groups with an interest in understanding and optimizing EHR transitions, as well as large-scale HIT implementations more broadly.

Previous studies about transitions from legacy (generally homegrown) to newer (generally commercial or vendor-based) EHRs [13,14,49,50] note that users frequently express a preference for the original EHR. In a few cases, authors briefly posit that this may be due to users’ loyalty to the older system or a perception that an EHR that has been in use for a long time is more likely than a newer product to reflect the health system’s workflows and practices [13,49]. However, none of the previous nonqualitative studies of EHR transitions assessed this issue, and the existing qualitative studies did not explore it in depth. Our study supports these previous authors’ conclusions and, furthermore, describes *why* employees may perceive a misfit between the new EHR and the health system’s needs, even a year following the new system’s implementation.

While acknowledging that a new EHR does not need to be perfectly tailored, the frontline employees we followed in our evaluation experienced the new EHR as a poorly tailored tool that made their work in VA cumbersome, inefficient, or downright impossible, when compared with the homegrown system. VistA /CPRS has been in continuous use in VA for almost 30 years, undergoing numerous changes to tailor it to VA’s specific needs at the organization and local site level. These needs reflect VA’s unique characteristics: complex and heterogeneous work involved in caring for individual veterans, caring for a population with generally more complex medical and behavioral needs than the nonveteran population [51,52], robust interprofessional communication and coordination, and, last but not the least, mission-driven emphasis on both patient-centered care and population health management [47,53,54]. For end users, transitioning to a vendor-based system has thrown into sharp relief both the benefits of a highly tailored legacy system and the challenges of transitioning to a commercial-based EHR. Focusing broadly on the challenges of replacing legacy software with commercial products in the public sector, a group of United Kingdom–based researchers argued that such transitions are exceptionally difficult because government agencies are risk-averse, burdened by bureaucratic structures, and excessively eager to hold on to their established work models [55]. This framing, however, implicitly assumes the older approach, congruent with the legacy software, to be inadequate and the newer manner of working, embodied in commercial technology, as preferable. The VA experience highlights a different possibility: What if, instead of introducing business-driven innovation, a commercial product disrupts the very elements of the organizational culture that are valuable and worth maintaining?

As we write, the future of VA’s EHR transition is surrounded by uncertainty. In October 2022, in the wake of critical reports by the VA’s Office of Inspector General about the effects of MGVAMC’s EHR transition on patient care issued in the preceding months [56-58], VA announced an extended pause in the EHR transition. Summer 2023 was suggested as the provisional date when the transition might resume, assuming that issues with the system’s functionality and safety are addressed [59]. In January 2023, a bill (HR608), “To Terminate the Electronic Health Record Modernization Program of the Department of Veterans Affairs,” was introduced in Congress. The following month, VA pushed back the EHR transition date at the next planned VA site, Saginaw Healthcare System in Michigan, to late 2023 or early 2024 [60]. On its end, Oracle Cerner indicated that it was looking to improve the training at future sites by signing a contract with Accenture & company that had been working with the DoD on its EHR transition [61]. However, in April 2023, as the previously stated deadline for resuming the EHR transition drew closer, VA issued a statement that no subsequent VA sites will go live with Oracle Cerner until safe use of the EHR system can be ascertained in measurable manners, with no specific dates provided [62]. One exception to that decision was the James A Lovell Federal Health Care Center in North Chicago, Illinois, which is an integrated VA and DoD health care system. Lovell Federal Health Care Center went live in March 2024.

If the EHR transition is resumed on a broader scale, it may be worked out in several distinct manners. First, it is possible that the VA and vendor will succeed in finding solutions for modifying the new EHR to better fit the VA’s practices and culture. Alternatively, we may see VA’s norms, workflows, and routines gradually change to fit the nonmodifiable features of Oracle Cerner. This could have difficult-to-anticipate consequences; it remains to be seen how such changes would impact VA’s mission as a veteran-centered system and a model of innovation and patient-centeredness. Finally, we could encounter a “holding pattern,” with employees developing and relying on workarounds to bridge the gap between the older manner of working and the new EHR. Like the previous scenario, this situation is also rife with uncertainty; literature has shown that while some workarounds may endanger the safety of clinical care, others may ultimately help *improve* existing workflows by offering creative alternatives to established processes and highlighting gaps in those processes [63,64]. It is indeed possible that *all* 3 scenarios may play out, depending on the specific issue at hand and the degree of leverage that each side exercises at a given moment.

Moving forward, it is essential that researchers continue to trace the dynamics and consequences of this mutual adjustment. VA has already created an infrastructure for ongoing research on EHR modernization, with plans to expand it in the near future [65,66]. Even if the transition to a new EHR were to be terminated, this would still present profound challenges. VistA/CPRS would need to be updated to address the issues that Oracle Cerner was contracted to address in the first place. Alternatively, if a different EHR vendor is contracted, this new relationship would also need to move forward while being mindful of the challenges encountered during the current transition. Any of the paths forward will present a classically wicked problem requiring “nuance, negotiation, and care” [67].

Beyond VA’s context, our findings have several important implications. First and most fundamentally, they reinforce an insight that is commonplace in social scientific studies of technology yet deserves wider recognition in health informatics and change management circles. Namely, EHR systems, like any IT product, are not neutral instruments—instead, they embody specific norms, values, and assumptions [68-70]. EHR transitions, therefore, are not a trivial matter of adjusting to an unfamiliar interface or learning a new manner of completing tasks—they are large-scale sociotechnical transformations [67] that may have a profound impact on the entire system of institutional practices, routines, and norms. The scope and complexity of the change may be even more pronounced for health systems that are preparing to replace their homegrown EHR with a commercial product.

While it may not be possible to anticipate all the wide-ranging and disruptive consequences of the EHR transition ahead of time, organizational leadership must understand that a large-scale, possibly prolonged disruption *will* happen. The literature shows that when users interact with an information system over time, they start to take its features for granted [68,70,71]. In the context of our study, it appears that the users *may*, in fact, become aware of the valuable characteristics of the legacy product because of having to adjust to a less well-tailored system. In such situations, users may struggle with the transition to a new product more than they would if the older EHR had also been vendor-based with limited customization at the organization and site level. It is essential that leadership clearly and proactively communicates the rationale behind adopting a product that may be less well-tailored to the organization’s perceived needs in some ways, providing a realistic picture of both challenges to come and benefits to be reaped.

Another implication of our findings is that health-system leaders would do well to take stock of how their organization’s values, principles, and priorities had been built into the older EHR. This insight is echoed by a recent case study of digital transformations in several European public-sector settings, which found that implementation was easier when an effort was made to understand the legacy system—its technical *and* cultural aspects alike [72]. Building on this inference, we also propose that health systems must consider how to optimize the fit between the new EHR and the organizational culture. Which features of the organization’s approach to care are nonnegotiable and must be reflected by the new EHR, and which ones can be changed if the new EHR functionality demands, so would be an essential distinction to make. Using a robust theoretical framework—for example, attending to which affordances (possibilities for action) are enabled or constrained by the interaction between the new versus old EHR and the health care organization’s specific context [10]—can be a promising direction. Leadership may also consider innovative tools and approaches informed by complex systems science and human factors engineering, such as human-in-the-loop simulation and tabletop exercises [73-75]. If modifying the organizational routines, practices, and values to enable the use of the new EHR is inevitable, the benefits of such changes must be clearly communicated to end users.

Finally, we advise that leadership leverage the in-depth organizational knowledge and first-hand experiences of end users to inform and improve homegrown-to-vendor EHR transitions. Here, learning health systems, like the VA, may have a head start given their long-standing history of involving clinicians first-hand in innovation and quality improvement initiatives, including informatics projects [76-78]. In VA, in particular, there are tremendous and largely yet-untapped opportunities for peer learning and support across sites as the multiyear EHR transition continues to move across the country. The community of practice [79] model, where sites can share knowledge, experiences, and support on an ongoing basis, may be particularly relevant for systems where a “rolling-wave” approach is adopted. Admittedly, the prospect of tailoring the new EHR to better meet the organization’s needs may pose a challenge if the organization is *also* confronted with the need for greater standardization [80], or if limited organizational resources are available for such tailoring. Future research should shed light on how various health systems navigate this dilemma.

### Limitations

Due to the use of snowball sampling for recruitment, employees who volunteered to participate constitute a self-selected subset that may not be representative of the site’s staff, and it is possible that alternative perspectives on the subject matter were not captured. However, as noted, the findings are generally consistent with the results of a survey with a large sample conducted as part of the same mixed methods project, strengthening the trustworthiness of the findings.

### Conclusions

VA’s transition from a highly tailored, homegrown EHR system to a vendor-based one is a high-profile development important in its own right and relevant for health-system leadership, EHR vendors, researchers, and other groups with an interest in understanding and optimizing EHR transitions and large-scale HIT implementations more broadly. We found that this transition has been not only a logistical challenge but a sociotechnical transformation that has had a profound impact on organizational culture and practices, perceived by many as undesirable and fraught with unintended consequences. The perceived lack of fit between the new EHR and VA’s institutional mission is a particular source of misgivings. In any health-system seeking to replace a highly tailored EHR with a different product, leadership must anticipate and proactively communicate about the disruptive nature of the transition, systematically assess and strive to improve the alignment between the new EHR’s functionalities and the organization’s needs, and leverage the organizational knowledge and insights of users at all levels during the implementation process.

## Appendix

Multimedia Appendix 1 COREQ (Consolidated Criteria for Reporting Qualitative Research) checklist.

Multimedia Appendix 2 Interview guides.

## Abbreviations

COREQ: Consolidated Criteria for Reporting Qualitative Research
CPRS: Computerized Patient Record System
DoD: Department of Defense
EHR: electronic health record
HIT: health information technology
IT: information technology
MGVAMC: Mann-Grandstaff Veterans Affairs Medical Center
VA: Veterans Affairs
VHA: Veterans Health Administration
VistA: Veterans Health Information Systems and Technology Architecture

## Data Availability

The data sets generated and analyzed during this evaluation are not publicly available due to the fact that the data for this work consists of in-depth interviews with Veterans Health Administration (VHA) employees conducted at a single, nonanonymous site. Due to the highly specific contextual information they contain, the transcripts cannot be more anonymized. Because of the sensitive nature of the subject matter and concern about participant privacy, we are unable to share our data set.
