# Supplementary material for: User Experiences of Transitioning From a Homegrown Electronic Health Record to a Vendor-Based Product in the Department of Veterans Affairs: Qualitative Findings From a Mixed Methods Evaluation
Source: JMIR Form Res. 2024 Sep 10;8:e46901. doi: 10.2196/46901 (PMC11422731; doi:10.2196/46901)
Supplement: Multimedia Appendix 1 [file formative_v8i1e46901_app1.docx]

**Multimedia Appendix 1: COREQ checklist**

Note: Where applicable, we indicate the section(s) of the manuscript wherein the relevant issue is discussed. In some cases, the pertinent information is too detailed/granular to have been included in the main text. Therefore, we provide the discussion in this checklist.

| Domain and items | Discussion |
| --- | --- |
| **Domain 1: Research team and reflexivity** | |
| Personal characteristics | Our multisite, multidisciplinary team of VA researchers came together in late 2019 after receiving funding to evaluate the upcoming electronic health record (EHR) transition planned by the Department of Veterans Affairs (VA). Our team consists of clinician and non-clinician health services researchers. All of us are employed by the U.S. Veterans Health Administration – a learning health system charged with providing healthcare and other services to Veterans, itself a branch of the VA. The team is co-led by a practicing physician-researcher (STR) and a senior qualitative methodologist (GS).  This manuscript’s co-authors constitute a subset of the larger team. Clinical backgrounds represented among the team members co-authoring this manuscript include internal medicine (JO), pulmonology (STR), speech-language pathology (MM), and psychology (GS). Non-clinical areas of expertise among the team members co-authoring this manuscript include anthropology (EA), qualitative research (EA, GS, JB, MM, SB), human-centered design (MM), implementation science (EA, CH), and informatics (STR, JB).  The origins of the evaluation that this manuscript draws on can be directly traced to the keen interest that we, as VA employees, had in understanding and improving the massive organizational transformation that VA’s EHR transition was anticipated to be. We were particularly invested in improving the experiences of frontline employees, noting with concern that no other evaluation efforts at the time focused on this important topic. However, we do not believe that our positionality as VA employees has biased our findings in any significant ways. As researchers, we are committed to rigorous data collection practices, as well as to reporting our findings in a truthful and transparent fashion, regardless of political expediency considerations. |
| Relationships with participants | None of the team members participating in recruitment and/or data collection efforts had any relationships with participants prior to the beginning of the evaluation. As described below, however, we gained initial access to the evaluation site leadership via introductions from our operational partner at the time – VA’s Office of Electronic Health Record Modernization (OEHRM).  During recruitment, our team shared aims of the evaluation (to understand and improve the experiences of VA’s frontline employees during the EHR transition), as well as general information about our locations within the VA system and professional backgrounds.  At the beginning of an interview with a new participant, each interviewer shared their professional background– for example, revealing lack of clinical background and/or lack of informatics expertise – to prompt the participant to offer clarification on pertinent clinical and/or informatics topics. No personal information was shared. However, as interviewers often had an opportunity to interview the same person 2-5 times over a period of a year or more, this did result in a strong personal rapport with their interviewees. |
| **Domain 2: Study design** | |
| Theoretical framework | We did not anchor our evaluation in a single theoretical framework due to the pragmatic nature of this project and the need for flexibility in responding to the developments in the field. Methodologically, we have from the beginning endeavored to follow the rapid learning cycle approach in the sense of tailoring our data collection and analysis efforts to the developments on the ground, as well as questions and concerns of our operational partners. Furthermore, the first and senior authors of this manuscript have been deeply influenced by social constructionism (EA) and grounded theory and phenomenology (GS), which may have shaped the interpretation and presentation of fundings. |
| Participant selection | Participant selection (snowball sampling) and the sample size are described in the Recruitment and Data Collection subsection of the Methods section of the manuscript.  It should also be noted that our team made every effort to recruit a broad array of participants across provider and support staff roles, as well as in multiple departments of the site, to ensure that diverse perspectives are represented appropriately. |
| Setting | The setting for data collection is described in the Recruitment and Data Collection subsection of the Methods section of the manuscript. It should also be noted that all interviewees were in the location of their choosing (workplace or home) during the interview; information about the location was not elicited.  A description of the sample is provided in the Recruitment and Data Collection subsection of the Methods section of the manuscript. We would like to emphasize that our sample includes individuals from a variety of roles (leaders and frontline employees, clinicians and support staff, clinicians from various disciplines, informaticists), which enhances the diversity of represented perspectives. |
| Data collection | Information on the interview guide development, repeat interviews, method of recording, field notes, an interview duration is provided in the Recruitment and Data collection subsection of the Methods section of the manuscript. The interview guides are attached with the manuscript. The following additional information is pertinent:  Transcripts were not returned to participants for comments and/or correction. This is not common practice in VA health services research. Furthermore, doing so would have impinged on the extremely limited time of our participants.  The information on saturation is available in the Data Analysis subsection of the Methods section. |
| **Domain 3: Analysis and findings** | |
| Data analysis | Descriptions of the analytic approaches (inductive/deductive content analysis and reflexive thematic analysis), software used, coding process and theme development is provided in the Data Analysis subsection of the Methods section of the manuscript. |
| Reporting | For each quotation in the manuscript, we provide an anonymized participant ID to indicate heterogeneity of exemplar quotes. We strived to ensure that the data presented and the findings are consistent. Further, we include both major themes and minor themes or discussion of diverse cases. |
